# Phase separation in biology; functional organization of a higher order

**DOI:** 10.1186/s12964-015-0125-7

**Published:** 2016-01-05

**Authors:** Diana M. Mitrea, Richard W. Kriwacki

**Affiliations:** Department of Structural Biology, St. Jude Children’s Research Hospital, Memphis, TN 38105 USA; Department of Microbiology, Immunology and Biochemistry, University of Tennessee Health Sciences Center, Memphis, TN 38163 USA

**Keywords:** Membrane-less organelles, Phase separation, Multivalency, Stress response, RNA metabolism

## Abstract

Inside eukaryotic cells, macromolecules are partitioned into membrane-bounded compartments and, within these, some are further organized into non-membrane-bounded structures termed membrane-less organelles. The latter structures are comprised of heterogeneous mixtures of proteins and nucleic acids and assemble through a phase separation phenomenon similar to polymer condensation. Membrane-less organelles are dynamic structures maintained through multivalent interactions that mediate diverse biological processes, many involved in RNA metabolism. They rapidly exchange components with the cellular milieu and their properties are readily altered in response to environmental cues, often implicating membrane-less organelles in responses to stress signaling. In this review, we discuss: (1) the functional roles of membrane-less organelles, (2) unifying structural and mechanistic principles that underlie their assembly and disassembly, and (3) established and emerging methods used in structural investigations of membrane-less organelles.

## Background

Similar to the division of labor in human societies, the cellular “workforce”, macromolecules such as proteins, DNA and RNA, is spatially organized in the cell based on functional specialization. Subcellular organization of macromolecules underlies vital cellular processes such as development, division and homeostasis, while disruption of this organization is often associated with disease.

A large proportion of the enzymatic and signaling reactions in biology occurs in aqueous solution. Lipid bilayers, immiscible with the aqueous phase, enclose the water-soluble components of a cell. The plasma membrane engulfs all the internal components of a cell. Membrane-bounded organelles provide the physical separation required for specialized processes to occur in functionally optimized compartments within a cell. Thus, the nucleus contains the machinery dedicated for DNA and RNA synthesis, while the cytoplasm houses components that control protein synthesis and degradation. The endoplasmic reticulum, Golgi apparatus and the lipid vesicles are membrane-bounded compartments specialized in protein sorting and trafficking through the cell. Mitochondria supply the ATP energetic needs of a cell, and are enclosed in a double layer membrane, in contrast to the single lipid bilayer that surrounds the other membrane-bounded organelles.

With the advent of electron microscopy that allowed visualization of nanometer scale structures [1] and advances in fluorescent dyes and light microscopy, it became evident that there is further sub-division and local organization within the nucleus and cytosol in the form of non-membrane bounded, macromolecular assemblies.

Currently characterized membrane-less bodies or organelles range in size from tens of nm to tens of μm and were defined as highly dynamic macromolecular assemblies, whose components rapidly cycle between the organelle and surrounding milieu [2–7]. Nucleoli (reviewed in [8]), nuclear speckles (reviewed in [3, 9]), paraspeckles (reviewed in [2, 10]), and PML (reviewed in [11, 12]) and Cajal bodies (reviewed in [4]) are enclosed within the nuclear envelope and are specialized in various aspects of gene regulation and RNA metabolism. Cytoplasmic messenger ribonucleoprotein (mRNP) granules, such as P-bodies, germ granules, and stress granules (reviewed in [13]) fulfill specific roles in mRNA metabolism and homeostasis. Analogous forms of RNA granules have recently been identified in mitochondria with roles in mitochondrial ribosome biogenesis and RNA processing [14].

In this review we will present an overview of current knowledge regarding the structural biology of membrane-less organelles and the molecular mechanisms involved in regulating their structure and function.

### Overview of membrane-less organelles

Membrane-less organelles were described as dynamic structures which often display liquid-like physical properties [5, 6]. Although it is well established that they are implicated in important biological processes, their precise roles remain elusive, often being associated with more than a single functional pathway. As will be described in greater detail in the following sections, the proteinaceous composition of membrane-less organelles and their morphology are altered in response to changes in the cellular environment. This ability to respond to environmental cues may represent the mechanistic basis for the involvement of the membrane-less organelles discussed herein in stress sensing [2, 4, 9, 11, 13, 15]. The lack of a lipid-rich barrier to enclose the constituents of membrane-less organelles presents the advantage that changes in the surrounding environment can readily alter their internal equilibrium. Release or sequestration of constituent proteins or RNAs from or within membrane-less organelles alters their concentrations in the surrounding freely diffusing pool of macromolecules, thereby sending signals that impinge upon stress response pathways. One example is the accumulation into the nucleolus, followed by release into the nucleoplasm of the tumor suppressor p14^ARF^ in response to DNA damage, which activates the p53 tumor suppressor pathway [16]. The nuclear volume is partitioned into multiple membrane-less organelles, also called nuclear bodies. Cytoplasmic bodies further partition the cytosolic components. Nuclear and cytoplasmic bodies are dynamic structures, with well-defined compositions, which have the ability to exchange components in response to alterations to their environment. In the following section we will discuss the functional roles of membrane-less organelles and the unique features that define them.

### Nuclear membrane-less bodies

#### The nucleolus

The largest and best studied membrane-less organelle, the nucleolus, functions as the center for ribosome biogenesis in eukaryotic cells. The nucleolus exhibits complex, compartmentalized organization in interphase and disassembles in mitosis. Three distinct regions can be observed by transmission electron microscopy (TEM) in intact nucleoli: the fibrillar centers (FC), dense fibrillar component (DFC) and granular component (GC). During mitosis, the GC dissolves, disrupting nucleolar organization but components of the FC and DFC maintain interactions as diffusible sub-structures.

Nucleolar assembly (reviewed in [8]) is initiated by RNA Polymerase I (RNA Pol I) transcription of clustered ribosomal RNA (rRNA) genes (rDNA) bound to the transcription factor UBF. Ribosome biogenesis occurs vectorially, starting from the FCs, where rDNA is transcribed into rRNA. pre-rRNA molecules transit through the DFC, where they are spliced and the small ribosomal subunit is assembled, then move into the GC where the large ribosomal subunit is assembled. Pre-ribosomal particles are then released into the nucleoplasm and subsequently exported into the cytoplasm where functional ribosomes are assembled.

p53-dependent stress sensing mechanisms are integrated into the nucleolus, thereby allowing the cell to halt the energetically expensive process of ribosome biogenesis under conditions that are unfavorable for growth and proliferation. For example, in response to oncogenic stress (e.g., activation of Myc), Mdm2, the E3 ubiquitin ligase responsible for rapid turnover of p53, is immobilized in the nucleolus through interactions with p14^ARF^ in order to upregulate p53 and its downstream cell cycle arrest effectors [17].

#### Paraspeckles

Paraspeckles are nuclear bodies located in the interchromatin space, with roles in control of gene expression through nuclear retention of specific RNA molecules, marked by adenosine-inosine editing [2]. The proteins that comprise paraspeckles are associated with RNA Polymerase II (RNA Pol II) transcription and processing of RNA. The DBHS family of splicing proteins, P54NRB/NONO, PSPC1, PSF/SFPQ [2, 10, 18, 19], and the long non-coding RNAs (lcnRNA) *NEAT1/Men ε/β* and *Ctn* are integral components of paraspeckles [2]. Paraspeckles are responsive to stress and exchange components with the nucleolus in response to environmental cues. For example, paraspeckle protein 1 (PSPC1) was first identified as a nucleolar protein; however, it was later shown that, under conditions of active RNA Pol II-dependent transcription, it partitions into a different nuclear body, the paraspeckles, and only becomes re-localized to the nucleolus when RNA Pol II activity is suppressed [10, 18]. Interestingly, this re-localization occurs at the peri-nucleolar caps, which are structures that appear to be physically associated with nucleoli, but are not integrated into the nucleolar matrix [10]. This suggests that either the physical properties of PSPC1-containing bodies and of the nucleolus are different, precluding fusion, or their dynamic behavior is restricted in response to the signals that inhibit RNA Pol II activity.

#### Nuclear speckles

Similar in appearance to paraspeckles and localized adjacent to nucleoplasmic interchromatin regions [3], nuclear speckles, also referred to as snurposomes, are a distinct class of dynamic organelles [1]. The composition of nuclear speckles, enriched in pre-mRNA splicing factors, such as small nuclear ribonucleoproteins (snRNPs) and serine/arginine-rich (SR) proteins [20], and poly(A)^+^ RNA [21], as well as their spatial proximity to sites of active transcription, suggest they may play a role in regulating gene expression by supplying or storing factors associated with the splicing of pre-mRNAs [22].

#### Cajal bodies

Although not fully elucidated, the role of the Cajal bodies is linked to regulation of snRNPs and small nucleolar ribonucleoprotein particles (snoRNPs) [4]. Time lapse experiments monitoring fluorescently tagged coilin and survival of motor neurons (SMN) proteins, two well described markers of Cajal bodies, showed that they are dynamic structures within the nucleus that undergo fusion and fission events [23]. Similar to other nuclear membrane-less organelles, Cajal bodies are responsive to stress conditions. The tumor suppressor p53 associates with Cajal bodies under conditions of UV-irradiation and chemotoxic stress [24], while coilin re-localizes to nucleolar caps, along with fibrillarin and components of the RNA Pol I machinery [25]. Furthermore, similar to the nucleolus, the structural integrity of Cajal bodies is cell cycle dependent; they are intact during interphase and dissolve during mitosis [26].

#### PML bodies

Localized primarily in the nucleus, PML bodies are characterized by the presence of promyelocytic leukemia (PML) protein. A member of the TRIM family of proteins, PML contains a RING domain, two B-box domains and a predicted coiled-coil domain, all of which have been shown to be required for proper assembly of PML bodies. The exact role of these organelles is yet to be fully elucidated. Evidence that transcriptional regulators such as p53, CBP and Daxx are transiently targeted and retained in PML bodies suggests that they function as a storage compartment and thus regulate pathways involved in tumor suppression, viral defense and apoptosis [12]. As with other membrane-less organelles, the number and structural integrity of PML bodies are influenced by cell cycle phase and stress stimuli [27]. In senescent cells, PML bodies become enlarged and associate with the nucleolar caps [28]. Newly synthesized RNA accumulates at the periphery of PML bodies, supporting a role in RNA metabolism. However, unlike the other membrane-less organelles described herein, RNA is dispensable with respect to the formation of PML bodies [29].

### Cytosolic membrane-less bodies

Dynamic membrane-less organelles were also described in the cytoplasm. They are generally referred to as mRNP granules, are involved in mRNA metabolism and homeostasis, and include structures such as P-bodies, stress granules and germ granules (reviewed in [13, 30]). Several different types of mRNP granules share protein and mRNA components and it has been demonstrated that they have the ability to physically interact with one another *in vivo*, undergoing docking and fusion events [13]. These observations suggest that not only are these membrane-less organelles functionally related, but under certain conditions they exhibit similar physico-chemical properties that allow for their structural miscibility. The major types of mRNP granules are discussed below.

#### P-bodies

Processing or P-bodies are ubiquitous to all types of cells and contain proteins involved in mRNA transport, modification and translation (reviewed in [31]). Studies in yeast demonstrated that deletion of any single protein component was not sufficient to fully abrogate the assembly of P-bodies [32], but highlighted the importance of partner-specific interactions to the accumulation of a number of proteins into the organelle [33, 34]. For example, recruitment of the Dcp1 decapping enzyme to the organelle is mediated by interactions with its co-factor, Dcp2 [34], while Dcp2 directly interacts with the scaffold protein Edc3 [33, 34]. As with other membrane-less organelles, RNA plays a central role in the assembly of P-bodies. Elevated levels of non-translating mRNA, achieved by inhibition of translation initiation or stress, is correlated with an increase in the size and number of P-bodies [35]. Conversely, entrapment of mRNA into polysomes by inhibiting the elongation step or enzymatic degradation of mRNA correlated with dissolution of P-bodies [31, 35].

#### Stress granules

Stress granules, as the name suggests, assemble in response to stress signals to sequester transcriptionally silent mRNA molecules and transcription factors (reviewed in [30]). Translation initiation factors and components of the small ribosomal subunit are amongst the proteins enriched within stress granules [13]. Removal of the stress signals and re-initiation of mRNA translation caused stress granules to disassemble [36]. Similarly to P-bodies, sequestration of non-translating mRNA molecules in polysomes inhibited formation of stress granules [36], thus suggesting that mRNA is required in their assembly. P-bodies and stress granules in yeast exhibit extensive compositional overlap, but distinct physical properties [37]. Furthermore, yeast strains deficient in formation of P-bodies were also unable to efficiently form stress granules. The formation of P-bodies in yeast was not affected in mutant strains that were deficient in stress granules assembly. Together, these observations suggested that pre-assembly of mRNA/protein complexes in P-bodies is a pre-requisite for the formation of stress granules [32], highlighting a functional connection between the two types of membrane-less organelles.

#### Germ granules

The term, germ granules, encompasses a class of non-membrane bounded organelles found in the specialized germ cells that generate sexual cells upon meiosis in the developing embryo and are referred to as P-granules, germinal bodies or Nuage bodies, depending on the organism of origin (reviewed in [38]). Significant advances have been made in understanding both the biology and the biophysics of P-granules in the nematode, *C. elegans.* P-granules are enriched in mRNA, RNA helicases and RNA modifying enzymes and are involved in the post transcriptional regulation of mRNA in primordial germ cells [38]. For example, nos-2 RNA is asymmetrically segregated during *C. elegans* larval development [39]. P-bodies physically dock, but do not fuse with germ granules in *C. elegans* embryos. This physical association between the two types of organelles allows P-bodies to segregate within the germline blastomere, a property borrowed from the germ granules. Furthermore, these P-bodies that are associated with germ granules fail to undergo maturation into organelles that degrade mRNA [40]. Collectively, these observations exemplify how distinct physico-chemical properties preserve organelle integrity and suggest inter-organelle interactions as a novel mechanism for regulating function.

#### mRNP granules in neurodegenerative disease

Debilitating neurodegenerative diseases such as amyotrophic lateral sclerosis (ALS), multisystem proteinopathy (MSP) and frontotemporal lobar degeneration (FTLD) are characterized by formation of pathological mRNP inclusions and disruption of normal mRNA metabolism (reviewed in [41]). These pathological inclusions are formed through aggregation of proteins found in endogenous mRNP granules. Interestingly, many of the proteins associated with pathological inclusions contain a prion-like domain in their amino acid sequence, which promotes their assembly into amyloid-like fibrils. Several proteins known to localize within stress granules, including FUS [42], hnRNPA1 [43–45] and hnRNPA2 [43], were found in ALS-associated pathological inclusions. Interestingly, fibril formation by these proteins is promoted within the stress granule microenvironment, where high local protein concentrations are achieved [37, 42, 44, 45]. Furthermore, genetic mutations within the prion-like domains of these proteins known to be associated with ALS accelerated formation of amyloid-like fibrils and inhibited stress granule clearance *in vivo*, thereby disrupting mRNA homeostasis [41–44]. These findings suggest that the highly dense environment of mRNP granules facilitates fibril formation by the proteins noted above, especially when their aggregation propensity is enhanced by mutation. Further, these studies establish correlations between ALS-associated mutations in mRNP granule proteins, and heightened fibril formation and altered mRNA metabolism. Additional research is needed, however, to understand how these changes to mRNP granule structure and function are related to neuropathogenesis.

In the next section we will discuss the common physico-chemical features of membrane-less organelles and unifying mechanistic insights that describe their assembly into multicomponent dense phases.

### Common features of membrane-less organelles

A hallmark of the membrane-less organelles described above is that their composition and physical properties vary depending upon cellular factors such as cell cycle stage, growth stimuli and stress conditions. In addition, they exhibit dynamic structural features. Brangwynne and colleagues demonstrated that the nucleolus [5] and P-granules [6] exhibit liquid-like behavior *in vivo* and that this fluid organization arises from phase separation of their molecular components. This concept is supported by a growing body of evidence identifying proteins, sometimes co-mixed with nucleic acids, that phase separate *in vitro* into dense liquid-like [46–49] or hydrogel [50, 51] structures (reviewed in [52]). The proteins and nucleic acids are concentrated ~ 10-100-fold in the dense phase [46, 48], where they can reach concentrations in the millimolar range [53]; the dilute phase is maintained at the critical phase separation concentration. Experimentally, the two physical states, liquid and hydrogel, are distinguished by their ability to flow when their surfaces are subjected to shear stress. The liquid-like features of membrane-less organelles and *in vitro* phase separated protein and protein/RNA droplets, have been demonstrated based upon measurements of their viscoelastic properties [5, 6, 44, 47, 54, 55]. For example, liquid-like P-bodies [37] and P-granules [6] adopted spherical shapes in the cytoplasm that were governed by surface tension, and coalesced and fused into larger droplets that returned to spherical shapes. Additionally, P-granules became reversibly deformed when they encountered a physical barrier (*i.e.* “dripped” on the surface of the nucleus) [6]. In contrast, hydrogels do not exhibit flow under steady-state conditions [50, 51, 56]. Microrheology analysis indicated that liquid-like membrane-less organelles [5, 6] and protein and protein/RNA droplets prepared *in vitro* are characterized by high viscosity. Strikingly, the measured values for viscosity varies widely, over a range of three orders of magnitude, from ~ 1 Pa · s for P-granules to ~ 10^3^ Pa · s for nucleoli [5, 6, 47, 54, 55]. Although not necessarily a direct indicator of liquid-like behavior, macromolecules within membrane-less organelles ([7, 37, 44, 46]) and liquid-like droplets [42, 44, 46, 53, 55] recover after photobleaching on a timescale of seconds to tens of seconds. This indicates rapid exchange of molecules within the liquid-like phase, or with the surrounding milieu, when the object is photobleached in part or in full, respectively.

Membrane-less organelles exhibit compositions of varied complexity. For example, P-granules are comprised of approximately 40 proteins [57] while mass spectrometry has shown that human nucleoli contain a staggering ~4500 proteins [58]. Furthermore, the protein composition of membrane-less organelles can vary depending upon cellular conditions. Notably, the nucleolar proteome is significantly altered under stress conditions and the alterations are specific to particular forms of stress [59, 60]. These observations raise two important questions: (1) *how is the specific molecular composition of membrane-less organelles achieved* and (2) *how is their composition regulated in response to stress signals*? In the next section we address the molecular principles that underlie phase separation and the structural organization of membrane-less organelles. We also discuss current evidence that suggests how their dynamic structure and compositions are regulated.

### Structural and compositional features of proteins resident within membrane-less organelles

Results from knock-down and knock-out studies [32, 39, 61–63] showed that the structural integrity of several membrane-less organelles depends upon heterogeneous interactions amongst multiple components. Knock-down or genetic deletion of single proteins, such as NPM1 [61] or nucleolin [62] in the nucleolus or PGL-1 and PGL-3 [63] in germ granules, altered organelle morphology but did not prevent other, unaltered organelle components from assembling into punctate structures. These observations are consistent with redundancy of the sequence features of proteins found within various membrane-less organelles (Table 1).

**Table 1:** Protein and RNA composition of membrane-less organelles

Organelle	Biological role	Protein	Domains/Motifs	RNA
Nucleolus	Ribosome biogenesis in nucleus	Fibrillarin	RGG box [133]	rRNA [8]
		Nucleolin	RRMs; RGG box [67]	
Paraspeckles	Regulation of gene expression in nucleus	PSPC1	RRMs; Coil [2]	ncRNA *NEAT1* (*Menε/β*); *Ctn* [2, 19]
		NONO/P54NRB	RRMs; Coil [2]	
		SFPQ/PSF	RRMs; Coil [2]	
Nuclear speckles	Regulation of gene expression via storage of splicing factors	SRSF1	RRMs; RS [134]	Poly(A)^+^ RNA; lncRNA MALAT1 [3, 134]
Cajal bodies	Regulation of snRNP maturation	Coilin	Coiled-coil [23]	snRNA; snoRNA [4, 135]
		SMN	Coiled-coil [23]	
PML bodies	Regulation of transcription and protein storage	PML	Coiled-coil [12]	None [11, 29]
Germ granules	Regulation of mRNA translation in the cytoplasm of germ cells	GLH-1, GLH-2, GLH-4	FG [74]	Developmentally regulated maternal mRNAs (*nos-1, pos-1, mex-1, skn-1 , gld-2*) [74, 136]
		PGL-1, PGL-3	RGG [63]	
		DDX4	FG; RG [48]	
		LAF-1	RGG box [47]	
P bodies	mRNA processing and decay	Pdc1	HLM; Coiled-coil [49]	mRNA [31]
		Dcp2	HLM [49]	
		Edc3	LSm; FDF [49]	
Stress granules	Storage of translationally stalled mRNA and proteins of the translational machinery	FUS	RRM; RGG box; [G/S]Y[G/S] [50, 137]	Poly-(A)^+^ mRNA associated with PABP [30]
		hnRNPA1	RRM; RGG box; [G/S]Y[G/S] [50]	

#### Basic principles of phase separation by polymers; from chemical polymers to proteins

**Fig. 1:**
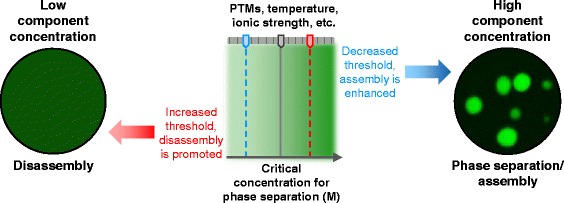
Macromolecular condensation mediates the formation of membrane-less organelles. Membrane-less organelles are dynamic structures formed via a polymer-condensation-like, concentration-dependent phase separation mechanism. The critical concentration threshold (*grey line*) for phase separation can be tuned within a range of concentrations (*shaded green box*) through physico-chemical alterations to the system (*i.e.,* posttranslational modifications to domains and/or motifs that alter the affinity of their interactions, changes in temperature, altered ionic strength, etc.). These changes can drive phase separation and assembly of membrane-less organelles, or their disassembly

Phase separation of organic polymers in solution has been extensively studied and can be described by simplified mathematical thermodynamic models. Flory-Huggins theory describes the free energy of mixing of a polymer with solvent, wherein polymers are treated as simplified arrays of modules that represent their repetitive segments. Liquid-liquid phase separation into a polymer-rich phase and a polymer-poor phase occurs when a critical concentration or temperature threshold is crossed, whereupon the polymer becomes a better solvent for itself than is the buffer it is dissolved in (reviewed in [64]; Fig. 1).

Rosen and colleagues reported that multivalent, repetitive domains from two signaling proteins that regulate actin polymerization, NCK and N-WASP, phase separate *in vitro* and that the phase separation threshold depends on the protein concentration and valency of each individual interaction partner [46]. Employing a simplified protein representation akin to that used for organic polymers, the authors used an adaptation of the Flory-Huggins formalism to describe the phase transition behavior of the binary NCK/N-WASP system. The model included four parameters: association/dissociation parameters, and diffusion and crowding coefficients. Qualitatively, this formalism, which assumed structural uncoupling between individual binding domains, predicted the effect of varying valency on the concentration threshold for phase separation [46]. A similar adaptation of this model was used to describe the phase separation behavior of the unimolecular RNA helicase, Ddx4 [48]. While the general phenomenology can be described using this simplified model, a recent report involving the binary NCK/N-WASP system demonstrated that charged residues within the disordered linker connecting SH3 domain binding modules caused weak self-association of NCK and reduction of the critical concentration for phase separation [65] (Fig. 1). Thus, Flory-Huggins theory describes the basic phase separation behavior of bimolecular and unimolecular protein systems. However, the sequence complexity of protein polymers, in contrast with compositionally more simple chemical polymers, provides the opportunity for additional inter-molecular interactions that can “tune” the phase separation phenomenon. These results provide a foundation for understanding the phase separation behavior of more complex systems *in vitro* in the future. Furthermore, they provide a foundation for in depth study of the behavior of membrane-less organelles in cells.

#### Protein elements associated with phase separation; low complexity sequences and folded domains

Proteins associated with membrane-less organelles often exhibit multivalent features which are manifested structurally in different ways. Folded domains are proteins segments which adopt discrete and stable secondary and tertiary structures. Disordered regions, also referred to as intrinsically disordered protein regions (IDRs), are protein segments that do not adopt stable secondary and tertiary structure and are conformationally heterogenous and dynamic. Some proteins within membrane-less organelles contain folded domains but may also contain IDRs, while others are entirely disordered (termed intrinsically disordered proteins or IDPs). A subset of disordered protein regions, termed low complexity regions, exhibit compositional bias towards a small set of amino acids. Interestingly, low complexity sequences and disorder [47, 48, 50, 56] are overrepresented in proteins shown to phase separate *in vitro*. These features provide a high degree of conformational flexibility which is required for binding events to remain uncoupled [46]. NMR analysis of proteins within the liquid-like phase after phase separation did not provide evidence of folding-upon-binding, thereby suggesting that the disordered low complexity regions preserve their conformational flexibility within the liquid-like phase [48, 53]. The detailed interpretation of these data is complicated, however, by the possibility for organizational heterogeneity of the protein molecules outside and possibly within liquid-like droplets, and the influence of inter-molecular interactions and apparent molecular size on resonance line widths and intensities.

**Fig. 2:**
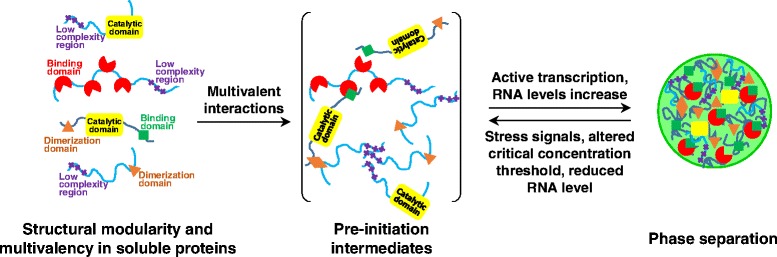
Molecular basis for membrane-less organelles assembly. The proteins enriched within the matrices of membrane-less organelles commonly exhibit multiple modules that create multivalency, including folded binding domains (*red*) and low complexity regions (*purple*). Valency is often amplified by domains that enable homo-, or hetero-oligomerization (*orange*). Interactions between proteins containing different combinations of these interaction modules provide a framework for building a heterogeneous, infinitely expandable network within membrane-less organelles. Formation of this type of network drives phase separation when the critical concentration threshold is reached. For many of the examples discussed herein, active RNA transcription is needed for membrane-less organelle assembly. We hypothesize that expression of RNA in excess of a critical concentration threshold is needed to nucleate interactions with specific, multi-modular proteins, and for nucleating formation of membrane-less organelles. Stress signals can alter the multivalent interactions that drive phase separation and lead to partial or complete disassembly of the organelle

Multivalent interactions are likely to contribute to the dynamic, liquid-like properties of phase separated unimolecular assemblies [47, 48], as well as of more complex assemblies [46, 49]. Amongst proteins associated with phase separation in membrane-less organelles, multivalency is achieved through repetitive display of two types of protein modules: i) folded domains and ii) low complexity disordered segments (summarized in Tables 1 & 2; Fig. 2). *In vitro* studies had shown that one of the two types of multivalency is necessary and sufficient for protein phase separation. The protein concentrations associated with phase separation varied over several orders of magnitude for different systems, ranging from sub-micromolar [44, 47] to hundreds of micromolar [44, 46, 48, 53]. Membrane-less organelles are multicomponent systems and their assembly, as demonstrated for the nucleolus, depends on the total concentration of their constituents [66]. Given the observations noted above that the accumulation of components with nucleoli is temporally defined (reviewed in [8]) and occurs at pre-formed nucleolar organizing regions (NORs) raises an important question. Are some components more important the others for initiating the phase separation process to form membrane-less organelles? Given the large differences in critical concentration measured for the various systems, one possible answer is that components with the lowest critical concentration phase separate first, thus increasing the local concentration above the critical concentration for phase separation of other components which subsequently become incorporated into the dense phase. Both folded domains and disordered/low complexity regions have been reported to initiate phase separation *in vitro* and *in cellulo.* The folded domains are often implicated in specific protein-nucleic acid [67–69] and protein-protein [19, 70] interactions and may provide an organizational scaffold for the assembly of a membrane-less organelle. Low complexity domains, on the other hand, provide a means for more dynamic interactions with a potentially broader range of binding partners (Fig. 2). A compelling example of such a synergistic cooperation between multivalent folded domains and their respective connecting flexible linkers was reported by Bajade et al., on the Nck/N-WASP/nephrin system [65]. Nck constructs that are divalent in SH3 motifs bind to PRM motifs in N-WASP with micromolar to millimolar  affinity and undergo phase separation. Through weak, largely electrostatically driven interactions, the disordered linker connecting the SH3 domains in Nck promotes self-assembly, effectively lowering the critical concentration for phase separation. Furthermore, addition of a disordered region of Nephrin containing multiple phospho-tyrosine residues, which bind to a folded SH2 domain within Nck, enhances multivalent interactions and further lowers the critical concentration for phase separation. Thus, multivalent display of folded domains and low complexity sequences with disordered regions within proteins enables synergy between the various components of complex liquid-like droplets. Similar synergy between multivalent components is likely to promote formation of membrane-less organelles in cells.

**Table 2:** Examples of protein regions involved in phase separation and their functional roles

Domains	Sequence/Structural features	Role
FG	FG/GFGG low complexity repeats	Association of P granules to the NPC [74]
RRM	Folded domain	RNA binding [19, 68]
Coiled-coil	Coiled-coil fold	Homo/hetero-dimerization [12]
RS	RS low complexity repeats	RNA binding; protein-protein interactions (Reviewed in [138, 139])
RGG	RGG low complexity repeats	RNA binding (Reviewed in [140, 141])
HLM	Short helical leucine-rich motif	LSm domains binding in P granules [49, 75]
SH3	Folded domain	PRM motif finding [46]
SH2	Folded domain	Phosphorylated tyrosine recognition [46]
PRM	Proline-rich short linear motif	SH3 domain binding [46]

#### Initiation events in the assembly of membrane-less organelles

Many of the proteins that participate in the formation of membrane-less organelles exhibit segments with low complexity sequence features, often containing multiple motifs enriched in the amino acids arginine, serine, glycine, glutamine, asparagine and/or aromatic residues (Tables 1 & 2). However, despite the low complexity of their sequences, these proteins are often associated with specific membrane-less organelles. What is the basis for the incorporation of particular proteins and nucleic acid molecules within particular membrane-less organelles? The emerging solution to this conundrum, at least in some cases, is that specific protein-nucleic acid or protein-protein interactions initiate the assembly of membrane-less organelles, which then create a microenvironment that is conducive to phase separation of additional components (Fig. 2). This concept was described for the nucleolus, which assembles around NORs, stable nucleolar precursors, comprised of clustered arrays (*i.e.* multivalency) of the genes for rRNA, bound to the transcription factor UBF [71]. Notably, UBF contains an array of six HMG box domains that exhibit a broad range of binding affinities for DNA [69]. RNA Pol I is recruited to the NORs to transcribe pre-rRNA, which initiates the assembly of the nucleolus. In the case of germ granules [63] and PML bodies [12], their formation is initiated by self-association of the coiled-coil domains of the proteins PGL-1/3 and PML, respectively. In these examples, structured domains mediate specific interactions to form assemblies that serve as scaffolds for further assembly of components of membrane-less organelles. Some of the proteins that promote assembly contain both structured domains and low complexity segments that mediate multivalent interactions. The formation of membrane-less organelles may thus involve hierarchical assembly of specific, higher affinity protein-nucleic acid complexes followed by the recruitment of additional components through weaker, multivalent interactions.

The assembly behavior of proteins associated with paraspeckles provides another example of how initiation events can mediate the recruitment of components within a membrane-less organelle. Bond and co-workers used X-ray crystallography and small angle X-ray scattering (SAXS) to study the polymerization of DBHS family of splicing factors, localized to and enriched in paraspeckles [19, 70]. Extended coiled-coil interaction motifs within the polymerization domain of these proteins provided the structural scaffold for formation of extended polymers of indefinite length. Weak, polar contacts stabilize the coiled-coil interactions and are thought to be advantageous in maintaining the solubility of unpaired extended helical structures [70]. The valency of the molecular assembly is enhanced by an additional dimerization domain which mediates homo- and hetero-dimerization between DBHS family proteins, such as PSPC1 and NONO [19] or SFPQ and NONO [70]. Furthermore, multivalent interactions with RNA are mediated by tandem RRM domains present in NONO, PSPC1 and SFPQ [19, 70]. These studies exemplify how modular, multivalent proteins can mediate the formation of heterogeneous, dynamic molecular assemblies, thereby providing the structural basis for formation of a membrane-less organelle (Fig. 2).

#### Forces that mediate the interactions associated with protein phase separation

As discussed above, proteins that undergo phase separation commonly contain segments with low sequence complexity. Further, these regions are often enriched in charged and aromatic amino acids, highlighting the importance of electrostatic and hydrophobic interactions in the process of phase separation. For example, disordered segments of the DEAD-box helicases Ddx4 [48] and LAF-1 [47], as well as hnRNPA1 [44] that mediate phase separation are enriched in arginine residues within their low complexity RGG box and RRM domains. Due to their overall positive charge, the formation of liquid-like droplets by these proteins is highly sensitive to the ionic strength of the surrounding solution. Numerous other proteins associated with nuclear bodies and mRNP granules are enriched in arginine residues (*e.g.* RGG and SR domains; see Table 1). For example, the low complexity SR repeats common to the SR family of splicing factors were identified as targeting signals for nuclear speckle localization [72, 73]. These observations strongly suggest that electrostatic interactions play a key role in the phase separation of a subset of proteins (Fig. 1).

Electrostatics are not, however, the only interactions that promote the formation of the protein-rich phase separated state. Low complexity regions that are rich in aromatic residues (*i.e.* phenylalanine, tyrosine) are overrepresented in proteins that reside within membrane-less organelles [48, 74] and other phase separated matrixes, as is the case for the FUS protein in mRNP granules [50, 53] and the FG-Nups in the nuclear pore complex [51]. Interestingly, mutations of F to Y, but not F to S, within the FG repeat domain preserved *in vitro* hydrogel formation by the yeast nucleoporin Nsp1p [51], demonstrating the importance of aromatic residues in assembly phenomena associated with the nuclear pore complex. Furthermore, the critical concentration for formation of *in vitro* FUS liquid droplets was lowered by increasing the ionic strength of the solution, consistent with the interpretation that salting out the hydrophobic interactions reduced the solubility threshold for the protein in buffer [53]. Nott et al., noted that evolutionarily conserved clustering of similarly-charged amino acid residues and regular spacing between the RG and FG motifs are required for the phase separation of a Ddx4 construct [48]. These studies highlight the roles of cation-π [48] and π-π [50, 51] interactions in phase separation phenomena.

In the absence of a lipid membrane barrier, the movement of molecules into and out of membrane-less organelles is diffusion limited [1], and their accumulation is mainly dependent on retention based on interactions with the organelle matrix. Interestingly, the diffusion barrier for exogenous macromolecules such as dextrans, is dictated by the physical properties of the membrane-less organelle matrix [1]. The DFC of the nucleolus is less permissive to accumulation of dextrans compared to the surrounding GC, consistent with the observations that the DFC is denser than the GC [1]. Furthermore, the dynamic features of components specifically retained within membrane-less organelles vary based on the nature of their interactions with other constituents of the matrix [7, 23]. Together, these results suggest that variable contributions of the different types of intermolecular interactions that promote phase separation determine selective accumulation of specific proteins within specific types of membrane-less organelles.

#### Mechanisms involved in achieving local organization and compositional complexity in membrane-less organelles

The localization of specific macromolecules within particular membrane-less organelles is achieved through specific interactions with the molecular network that extends from the nucleating region. As discussed above, a large proportion of the proteins known to associate with membrane-less organelles exhibit multivalency through the display of repeated low complexity motifs (e.g., SR, RGG or FG motifs) and/or of multiple copies of folded domains, such as RRM domains. Through combinatorial utilization of a finite number of intermolecular interaction modules, complex mixtures of proteins and nucleic acids can thus be recruited into the condensed phase. For example, the formation of P-granules is initiated by self-association of the coiled-coil domains of PGL-1 and PGL-3 proteins, which further bind mRNA via their low complexity RGG domains. Vasa-related helicases GLH-1, 2, 3 and 4 that contain FG repeats are then incorporated to facilitate P-granule association with nuclei, through interactions with and expansion of the nuclear pore complex hydrogel matrix [74]. The presence of homo- and hetero-oligomerization domains further enhances the degree of multivalency and promotes integration within membrane-less organelles (Fig. 2). The PML protein forms homo- and hetero-oligomers via its coiled-coil domain, but valency can be increased by homo-dimerization through the RING domain. Mutations in either the coiled-coil or RING domains led to disruption of PML bodies [12]. Components of the mRNA decapping machinery found in P-bodies, including Pdc1, Dcp2 and Edc3, assemble into liquid-like droplets *in vitro.* Two LSm domains in dimeric Edc3 interact with Dcp2 and Pdc1, which both contain multivalent HLM motifs. Edc3 binds to various HLM motifs with affinities within the low micromolar to millimolar range [49]. The valency of the HLM motifs in Pdc1 is increased through oligomerization via a central coiled-coil domain [49, 75]. These examples illustrate how multivalent interaction modules and oligomerization domains can cooperate to initiate phase separation in the context of different types of membrane-less organelles. Additional domains within these proteins, which are not directly involved in the mechanism of phase separation, can mediate the recruitment of additional components into the liquid phase. For example, the helicase Ddx6/Dhh1 and mRNA can be recruited to P-bodies via the FDF domain of Edc3 and the RNA binding domain of the helicase, respectively [49]. We thus distinguish between two basic types of components of membrane-less organelles: (i) multivalent macromolecules that directly participate in interactions involved in the process of phase separation and underlie the structural features of the liquid phase and (ii) other macromolecules that are recruited via specific interactions with the phase separated assembly, which lack multivalent interaction elements, but perform specialized functions within the liquid phase (*i.e.,* enzymes that catalyze specific biochemical reactions). However, the capability for assembly/phase separation and biochemical functionality can be embodied within a single protein, as is seen with Ddx4, which harbors a helicase domain and a multivalent, low complexity RGG domain that mediates phase separation [48].

#### RNA within membrane-less organelles

While much attention has been given to understanding the roles of multivalent proteins in the formation of membrane-less organelles, the primary functions of many of these organelles are different aspects of RNA metabolism and, consequently, RNA is also involved in their assembly and structural integrity. The assembly of the nucleolus at the exit of mitosis is initiated by transcriptional activation of RNA Pol I [8, 76] and the structural integrity of paraspeckles is dependent upon transcriptional activity of RNA Pol II [2]. Proteins capable of undergoing phase separation often contain similar sets of folded and low complexity multivalent domains, giving rise to structural redundancy and the potential, under certain conditions, to promiscuously localize within more than one types of membrane-less organelle. In contrast, the different types of organelles generally contain specific types of RNA (summarized in Table 1), suggesting that the RNA components are the principal determinants of organelle identity. In support of this hypothesis, disruption of RNA transcription causes re-localization of the protein components of different nuclear and cytoplasmic bodies [25, 59]. For example, Mao et al., demonstrated that the lncRNA Mem ε/β was required for the recruitment of specific protein and RNA molecules to paraspeckles [77]. Additionally, immobilization of PSP1, a modular, paraspeckle protein shown to homo- and hetero-oligomerize [18], was able to recruit some paraspeckle protein components, but was unable to recapitulate complete assembly of the organelle [77]. Recruitment of the full complement of protein and RNA components of paraspeckles, coupled with exclusion of macromolecules associated with nuclear speckles, was achieved only under conditions of active transcription of the Mem ε/β lncRNA. While the observations summarized above clearly indicate the dominant role of RNA in the molecular makeup of certain membrane-less organelles, other factors can also influence their structural integrity. For example, stress signals induced by DRB, a small molecule that selectively inhibits RNA Pol II, caused dissolution of paraspeckes before a significant decrease in the total Mem ε/β lncRNA levels could be measured [77]. This finding suggests that a currently unknown regulatory mechanism controls the structural integrity of paraspeckles and that there is a sharp and sensitive threshold for sensing and responding to cellular stress. This raises an important general question: *how are changes in environmental conditions, for example in response to different types of stress, transmitted to the membrane-less organelle matrix and manifested as changes in structure and function*? This topic is discussed in the next section.

### Structural and dynamic regulation of phase separated structures

The lack of a lipid bilayer barrier between membrane-less organelles and their surroundings circumvents the need for active transport of macromolecules across membranes and enables rapid signal transduction. Stress signals influence the structural integrity of membrane-less organelles, providing a mechanism for organelle-mediated stress responses. We next discuss various factors that influence the structure and function of membrane-less organelles.

#### Chemical and other environmental factors

Changes in temperature [27, 48], ionic strength [47, 48], and chemotoxic and DNA damage [27, 59, 60, 78, 79] are environmental changes known to disrupt phase separated cellular bodies and *in vitro* liquid droplets. The stiffness of nucleoli isolated from HeLa cells was decreased or increased upon RNA Polymerase or proteasome inhibition, respectively, based on atomic force microscopy measurements [79]. Thus, stress signals affect the viscoelastic properties of nucleoli and consequently modulate their functions.

Membrane-less organelles form, disassemble and function in an intracellular environment crowded with macromolecules. The high cumulative concentration of macromolecules in the cell, which correlates with a high percentage of excluded volume (~20–30 % of the total cell volume), affects the kinetics and thermodynamics of most biochemical processes [80]. *In vitro,* molecular crowding agents promote assembly of recombinant hnRNPA1 into protein dense liquid-like droplets at lower critical concentrations than observed in buffer alone [44, 45]. Thus, the increase in excluded volume caused by macromolecular crowding increases the local concentration of individual protein species, thereby decreasing the effective concentration threshold for phase separation (Fig. 1).

Alterations in the morphology and viscoelastic properties of mRNP granules, due to mutations in resident proteins (*e.g.* hnRNPA1, FUS) are associated with debilitating neurodegenerative diseases [13, 42, 44, 45]. *In vitro*, both FUS and hnRNPA1 phase separate into liquid-like droplets [42, 44, 45, 53] or hydrogels [42, 44, 50], depending on protein concentration and experimental conditions. The low complexity regions in the two proteins, along with the RRM domains [44, 45, 53], contribute to phase separation. Mutations within Q/N-rich low complexity regions, termed prion-like domains, are associated with defects in mRNP granules and neuropathogenesis [42, 44]. These defects are attributed to a kinetically slow step (tens of minutes to hours time scale) that occurs in the dense liquid-like phase, referred to as “droplet aging” [42], wherein the liquid-like phase transforms into a solid-like state. Phenomenological observations suggest that this physical transformation is a result of a slow structural re-organization of the dense, liquid-like phase. The reorganization leads to decreased dynamics within the phase separated state and culminates in a transition from a liquid-like state to a hydrogel or solid-like state. The transition between the two physical states is accompanied by morphological changes, from nearly spherical droplets, shaped by surface tension, to elongated, fibril-like structures [42, 44, 45]. A similar transition was observed *in vitro* and *in vivo* droplets containing Whi3, a protein encoding a polyQ tract [55]. A potential underlying mechanism is that under the conditions of the high local protein concentration within the dense, liquid-like phase, new, less dynamic interactions occur, perhaps between the low complexity prion-like domains. In time, these interactions may become dominant over the more dynamic, multivalent electrostatic interactions that give rise to the liquid-like state. We speculate that the balance of the thermodynamic favorability of these two types of interactions may influence the physical nature of the phase separated state (*i.e.,* liquid, hydrogel/solid) and determine the different propensities of wild-type and mutant proteins to undergo the transition for the liquid-like to solid-like structural state.

#### Energy-dependent control of membrane-less organelle dynamics

We have emphasized that the physical properties of membrane-less organelles depend upon their protein and RNA composition. In addition, however, the nucleolus requires ATP in order to maintain its liquid-like behavior, a physical state termed an “active liquid” [5]. It is currently unclear what specific ATP-dependent processes are involved in maintaining this active liquid-state. Furthermore, the activity of ATP-dependent chaperones, such as Hsp70/Hsp40, which accumulate within stress granules, is required for their disassembly upon recovery from stress [81]. These observations suggest that ATP-hydrolyzing enzymes regulate the dynamics of macromolecules within membrane-less organelles. Similarly, several other types of ATP-dependent enzymes, including kinases and DEAD-box helicases [47–49, 78], which are incorporated into these organelles, may be involved in maintaining their liquid-like physical properties. Helicases may modulate RNA structure as well as protein-RNA interactions and, thereby, actively control the viscoelastic properties of membrane-less organelles.

#### Role of posttranslational modifications in regulating membrane-less organelle structure and dynamics

The assembly of components within many of the phase separated systems we have discussed is electrostatically driven. Therefore, posttranslational modifications that alter the charge features of amino acids within the domains and low complexity segments of proteins provide a means to modulate their multivalent interactions and phase separation behavior (Fig. 1).

The importance of electrostatic interactions is illustrated by the phase separation behavior of LAF-1 [47], hnRNPA1 [44, 45] and Ddx4 [48], whose ability to form liquid-like droplets is strongly influenced by the salt concentration of the surrounding buffer. The phase separation concentration threshold for both scaled linearly with ionic strength as the NaCl concentration was increased. In addition, methylation of arginine residues in the RGG domain of Ddx4 increased the phase separation threshold *in vitro* [48].

Phosphorylation plays a crucial role in many signal transduction pathways and also modulates the structural integrity and dynamics of membrane-less organelles. For example, tyrosine phosphorylation of nephrin stimulates the phase separation of the ternary system nephrin/NCK/N-WASP [46]. Interestingly, a common feature of certain well-characterized membrane-less organelles is that they incorporate kinases and phosphatases within their matrixes [39, 78, 82]. Active phosphorylation/dephosphorylation cycles have been linked to regulation of organelle structural integrity. The activity of the nucleolar kinase CK2 controls the structural connectivity between the GC and the DFC regions within the nucleolus [78] and increases the dynamics of NPM1 exchange between the nucleolar and nucleoplasmic compartments [83]. Furthermore, phosphorylation of MEG-3 and MEG-4 proteins by MBK-2/DYRK kinase and dephosphorylation by PP2A^PPTR-1/PPTR2^ phosphatase regulates P-granule disassembly and assembly, respectively, during mitosis in *C. elegans* in association with embryogenesis [39].

Assembly and disassembly of membrane-less organelles provides a mechanism for controlling the concentration and associated signaling behavior of freely diffusing molecules within the membrane-bounded compartments of the cell. For example, the dynamic properties of stress granules are coupled with mTORC1 signaling by immobilization of mTORC1 within the granules, while phosphorylation-mediated dissolution of these organelles liberates mTORC1, activating downstream signaling [82]. As another example, Wippich et al. [82], demonstrated that the kinase DYRK3 condenses in cytoplasmic granules via its low complexity N-terminal domain, in a concentration dependent manner, and localizes to stress granules under osmotic and oxidative stress. Inactive DYRK3 condensed into stress granules, together with components of the mTORC1 pathway. Activation of DYRK3 and downstream phosphorylation of PRAS40, an mTORC1 inhibitor, results in dissolution of stress granules and disruption of the inhibitory PRAS40/mTORC1 interaction.

Further evidence for the role of posttranslational modifications in regulation of the features of membrane-less organelles is provided by the observation that the amino acids arginine, serine and tyrosine are overrepresented in the low complexity sequences of proteins within them. These amino acids can be posttranslationally modified, arginines by methylation and serines and tyrosines by phosphorylation, providing general mechanisms for modulating protein condensation thresholds and consequently the signaling pathways downstream of components sequestered within the phase separated fraction.

#### Component concentration as a factor in membrane-less organelle assembly/disassembly

Another important factor in phase separation-dependent formation of membrane-less organelles is the local concentration of components (Fig. 1). For example, regulation of P-granules during the oocyte-to-embryo transition, when they transit from the perinuclear region to the cytoplasm, is regulated by a concentration gradient, which causes dissolution of the perinuclear droplets and re-condensation in the cytoplasm. A similar mechanism is employed during the asymmetric segregation of P-granules into the germline founder cell [6]. Recently, Brangwynne and colleagues demonstrated that the levels of RNA in LAF-1 droplets, a minimalistic *in vitro* model of P-granules, tunes the viscosity and molecular dynamics within the liquid-like phase [47]. The viscoelastic properties of liquid-like droplets containing Whi3 are also modulated by RNA concentration. While Whi3 is able to phase separate in a unimolecular fashion under certain conditions, the presence of RNA is required for the process to occur at physiological salt concentrations. Furthermore, an increase in the RNA concentration correlates with an increase in droplet viscosity and a decrease in Whi3 dynamics of recovery after photobleaching [55]. In addition, assembly of nucleoli and paraspeckles depends upon the concentrations of their constituent RNAs, which are controlled by the transcriptional activity of RNA polymerases [2, 8], suggesting that transcriptional control of RNA concentration may be a general mechanism to tune the physical properties of membrane-less organelles (Fig. 1).

Many membrane-less organelles are involved in cellular responses to various types of stress and the sensitivity of their structural integrity to protein and RNA concentrations provides a mechanism for rapidly responding to stress signals that affect these levels. For example, inhibition of Pol I-, II- and III-dependent RNA transcription by Actinomycin D was associated with re-organization of constituents of both nuclear and cytoplasmic membrane-less organelles [59]. After Actinomycin D treatment, NPM1, a major component of the GC of the nucleolus, becomes delocalized to the nucleoplasm and cytoplasm due to inhibition of RNA Pol I-dependent transcription of rRNA. Under these conditions, cytoplasmic NPM1 was found to interact with components of stress granules, such as mRNA, and the proteins hnRNPU and hnRNPA1 [84].

Also under conditions of Actinomycin D treatment, protein and RNA components associated with paraspeckles, and PML and Cajal bodies, re-localize to nucleolar caps. Interestingly, while proteins from the GC are ejected from the nucleolus, proteins from the DFC, such as fibrillarin, re-localize to nucleolar caps [25]. These observations suggest that environmental changes can alter the equilibria that maintain the integrity of membrane-less organelles, thereby altering the concentrations of their components in the freely diffusing pools of macromolecules within the nucleoplasm and cytoplasm and allowing their redistribution within various other organelles.

### Emerging methods for the study of phase separated structures

Detailed analysis of the structural features of membrane-less organelles and their underlying macromolecular assemblies presents challenges not encountered in other areas of structural biology. Interactions relevant to the phase separation phenomenon occur over multiple length scales, from sub-nanometer to tens of micrometers, thereby making any single analytical technique insufficient for the study of phase separated macromolecular assemblies. For example, while liquid-like droplets exceed the size limitations associated with analysis by NMR spectroscopy, the structural and dynamic features of flexible components within them have been characterized [53]. However, the dynamic features of these systems are incompatible with X-ray crystallography. Although the macromolecular assemblies formed are readily observable by conventional microscopy techniques, the interactions responsible for assembly occur on length scales that are below the resolution limit of detection. Additionally, these systems are highly heterogeneous and therefore, integrative solutions that combine complementary methods are needed in order to understand their structural features.

#### Atomic-resolution structure determination methods

Several studies utilizing classical structural methods, including solution NMR [46, 48, 49, 67–69] and X-ray crystallography [19, 70], have provided detailed insights into the molecular interactions that mediate the network structure that drives phase separation of modular proteins within membrane-less organelles. However, due to technological limitations, these studies were performed with truncated forms of the proteins and nucleic acids corresponding to individual interaction modules. These traditional methods will be useful in the future for determining the structural basis of interactions between folded domains within multi-domain phase separation-prone proteins and their interaction partners, including peptides corresponding to short linear motifs and segments of RNA. However, because many phase separation-prone proteins exhibit low complexity and disordered sequence features, these methods for determining discrete protein structure are likely to receive limited application in this emerging field.

#### NMR spectroscopy; a versatile tool in studies of phase separation-prone proteins

NMR spectroscopy offers unique capabilities in studies of disordered proteins, by providing insights into conformations and dynamics of individual amino acids throughout the polypeptide chain. Measurements of chemical shift values for nuclei of backbone atoms report on secondary structure propensities and dynamics can be probed on ps to ns, and μs to ms timescales using a variety of relaxation methods [85]. Furthermore, long-range structure within disordered proteins can be studied using paramagnetic relaxation enhancement (PRE) methods and through the measurement of residual dipolar couplings [86]. The former method, however, requires that proteins be engineered to include single cysteine residues for labeling with a paramagnetic probe. A limitation of these NMR approaches is that rapid conformational fluctuations of disordered polypeptides causes ensemble averaging of NMR parameters. A second limitation is that the structural and dynamic information gained reports on the features of individual sites within a protein on a very limited length scale (Å or tens of Å in the case of PRE measurements). An exception is the use of pulsed field gradient methods to study protein diffusion [87] but this has not yet been used in studies of proteins within liquid-like droplets. The extensive dynamics that characterize IDPs are often an advantage for NMR studies because they cause resonance narrowing and enhance detection. However, some IDPs experience motions on time scales that cause resonance broadening and can hamper NMR studies. Despite these limitations, NMR has already been demonstrated to provide unique insights into the conformational and dynamic features of phase separation-prone IDPs both before and after phase separation; several exemplary studies are discussed below under “Integrative approaches to understand the molecular basis of phase separation”.

#### Methods to study molecular interactions associated with phase separation

Classical methods for characterization of biomolecular interactions, such as ITC [49] and SPR [68, 69], have been employed to characterize the wide range of binding affinities associated with the different types of interactions that occur within liquid-like droplets and/or membrane-less organelles. NMR can also be used to characterize macromolecular interactions and is particularly well suited in studies of weak interactions that present challenges for other methods. For example, chemical shift perturbations observed during titrations of an unlabeled binding partner into an isotope-labeled protein can be quantitatively analyzed to report residue-specific and global K_d_ values for interactions associated with phase separation [NPM1 integrates within the nucleolus via multi-modal interactions with proteins displaying R-rich linear motifs and rRNA: Mitrea DM, et al., under review]. However, the multivalent features of phase separation-prone proteins can give rise to complex, multi-step interaction mechanisms, which complicate the analysis of data from the methods discussed above. Therefore, experiments are often performed with truncated macromolecules of reduced multivalency and therefore do not address interactions under the conditions of phase separation. Despite these limitations, these biophysical methods provide important insights into the binding features of the individual elements within multivalent macromolecules that undergo phase separation.

#### Scattering methods to probe structural features before and after phase separation

Dynamic light scattering and small angle X-ray scattering (SAXS) [19, 46] have been employed to gain insight into the overall size and shape of the macromolecular assemblies. In particular, SAXS has been used to characterize the shapes (e.g., radius of gyration) of ensembles of disordered proteins [88]. However, scattering methods can also detect long-range order within so-called soft materials and uniquely provide insights into the structural makeup of these materials. Small-angle neutron scattering (SANS) has previously been employed in the structural analysis of polymer blends [89–91] and polymeric soft nanomaterials [92] and has great potential in studies of membrane-less organelles to provide information about the spatial organization of macromolecules within the condensed state. One recent study used SANS to characterize the regular spacing of molecules within droplets comprised of the nucleolar protein, nucleophosmin (NPM1), and a peptide derived from the ribosomal protein, rpL5, on length scales from 5.5 to 11.9 nm [NPM1 integrates within the nucleolus via multi-modal interactions with proteins displaying R-rich linear motifs and rRNA: Mitrea DM, et al., under review]. SANS has the advantage of allowing detection of scattering from specific components within heterogenous, phase separated states through selective protonation and/or deuteration and solvent contrast matching [93]. Furthermore, time-resolved SANS has been used in the past in studies of mutant huntingtin exon 1 phase separation into amyloid fibers to determine the mechanism of macromolecular assembly and the geometry of monomer packing within the fibrils [94]. We envision that SAXS and SANS may be able to reveal the spacing of partially ordered macromolecules within the liquid-like structure of droplets prepared *in vitro* and possibly within membrane-less organelles if technical issues associated with sample preparation can be addressed. We envision that these scattering methods will be powerful tools in the characterization of biological structures that arise from phase separation in the future.

#### Light microscopy

Light microscopy methods (reviewed in [95]) have been extensively utilized to visualize the subcellular localization of fluorescently tagged molecules. Live imaging coupled with fluorescence recovery after photobleaching (FRAP) or fluorescence loss in photobleaching (FLIP) methods probe the dynamics of macromolecules within membrane-less organelles inside living cells [7, 46, 48, 77] and phase separated states reconstituted *in vitro* [46–48, 50].

The information obtained from structural biology methods is on length scales of 10^−10^–10^−9^ m, while the classical light microscopy techniques provide information on much greater length scales, from 10^−7^ to 10^−3^ m. This situation creates a gap corresponding to two orders of magnitude on the length scale in our understanding of the structural and dynamic features of micron-sized membrane-less organelles. Macromolecular interactions that occur on the length scale of this gap are responsible for the structural organization that gives rise to phase separation and the liquid-like and/or gel-like properties of membrane-less organelles and related structures. We next discuss structural methods that can peer into this length scale gap.

#### High resolution and single-molecule microscopy

Electron microscopy can extend into the length scale gap between the two sets of techniques described above and has been extensively utilized to study cellular ultrastructure [1]. A significant limitation of this technique is the low certainty with which specific molecules can be identified based upon the greyscale contrast of images [96]. The emerging field of correlated light and electron microscopy (CLEM; reviewed in [96]) presents the opportunity of directly connecting dynamic information obtained via live fluorescence microscopy methods with ultrastructural detail acquired by electron microscopy.

Significant advances were made in the last decade in super resolution microscopy methods (reviewed in [97]) and were successfully applied to decipher chromosomal architecture [98]. Lattice sheet microscopy coupled with structured illumination microscopy, a method that returns 3D images with resolution ~ 200 nm x 200 nm in the x/z plane that exceeds the diffraction limit, was applied to study the ultrastructural organization of germ granules in *C. elegans* [39]. The internal structure observed in several membrane-less organelles suggests that the condensed macromolecules are not homogenously distributed, but further partition into phase separated fractions with distinct physical properties. These methods provide opportunities to reveal the heterogenous ultra-structure of membrane-less organelles in the future.

Single-molecule fluorescence microscopy holds great potential in the analysis of proteins within liquid-like droplets *in vitro* and membrane-less organelles in cells. For example, single-molecule fluorescence correlation spectroscopy (FCS) [99] and Förster resonance energy transfer (smFRET) [100] have been used to study the structural and dynamic features of aggregation-prone intrinsically disordered proteins *in vitro* (reviewed in [101]). In addition, single-molecule FRET and other methods have been applied to a wide range of disordered proteins with varied charged residue compositions and distributions (reviewed in [102]). We envision that these methods will be applied in the future to disordered proteins within liquid-like droplets to reveal their structural and dynamics features. Furthermore, smFRET and fluorescence lifetime imaging have revealed the conformational features of a disordered protein within HeLa cells [103], providing opportunities in the future for studies of phase-separation-prone proteins within membrane-less organelles in their natural cellular setting.

#### Additional physical characterization methods

Density [1], viscosity [5, 6, 47] and stiffness [79] are a few of the physical properties that have been measured for *bona fide* membrane-less organelles or *in vitro* reconstituted liquid droplets. Interferometer microscopy was utilized to measure the density of nuclear membrane-less organelles in isolated *Xenopus laevis* germinal vesicles, oocyte nuclei [1]. This method provided important insights into the physical properties of refractory sub-cellular bodies in a quasi-natural environment. A few considerations when interpreting these data, however, are that the results are based on the simplified assumptions that the organelles are spherical in shape and are exclusively composed of homogenously mixed water, proteins and low molecular weight solutes [1].

Atomic force microscopy provides the advantage of performing surface scans of membrane-less organelles which produce topological maps with resolution in the nanometer range. Also, this method provides a means to measure other key biophysical properties, such as structural stiffness, as done for nucleoli [79].

Microrheology methods, traditionally used in the characterization of viscoelastic properties of polymers and complex fluids [104], were applied to the characterization of membrane-less organelles [5, 6, 42, 105] and *in vitro* formed protein and protein-RNA liquid droplets [47, 55]. In particular, the tracer bead technology provided important insights into the effect of RNA onto the viscoelastic properties of *in vitro* liquid droplets [47, 55].

#### Computational and theoretical approaches

As we gain greater knowledge of the types of macromolecules that undergo phase separation to form liquid-like structures both *in vitro* and in cells, computational models are needed to analyze the structural and dynamic features, encoded by their amino acid sequences, so as to understand their phase separation behavior. A large proportion of the proteins, or protein regions, shown to undergo phase separation are intrinsically disordered, which presents a variety of computational challenges, notably conformational sampling and physical accuracy. A wide variety of methods are used to address the need to sample the extensive conformational space explored by IDPs/IDRs, including molecular dynamics methods, often enhanced by approaches such as replica exchange and related methods [106, 107], and Monte Carlo sampling methods [108, 109]. Many different force fields and variants thereof are available [110–112] and several were recently tested and compared [113]. Computations are often performed without experimental restraints and therefore they are reliant on the underlying force fields for generation of physically accurate molecular ensembles. A problem in the past was that computational models of IDPs were overly compact [114] but this issue is being addressed through the method refinement [112, 115–117] and consideration of NMR, SAXS and smFRET data [110, 113, 118]. Another group of approaches utilize experimental restraints (e.g., NMR and/or SAXS data) to select conformers for inclusion within IDP ensembles—the so-called “sample-and-select” methods [88, 119–121]. Complementary computational methods have been developed for generating IDP ensembles based on SAXS data [122]. The development of physically accurate molecular ensembles with atomistic detail for IDPs is important because, with the exception of single-molecule fluorescence methods, the experimental methods used to characterize IDPs are subject to ensemble averaging. Therefore, computationally generated ensemble models of IDPs enable examination of the features of large numbers of individual molecules. However, these approaches are only beginning to be applied to proteins that undergo phase separation.

A key challenge in computational studies of phase separation-prone proteins is to gain insight into the inter-molecular interactions that are the basis for self-association and phase separation. Regarding this goal, the field is in its infancy. However, methodologies applied to understand protein aggregation and fibril formation can be leveraged to understand the types of interactions that drive protein phase separation and possibly, in the future, protein-nucleic acid phase separation. In the protein aggregation field, course-grained computational methods have been applied to understand the aggregation of poly-glutamine tracts associated with Huntington's disease [123] and atomistic methods to understand aggregation of amyloid β [124]. Clearly, increased effort in this area is needed to understand the molecular basis for phase separation.

While computational approaches face challenges in addressing the protein phase separation problem, significant progress has been made in recent years in understanding relationships between the sequence features of IDPs and IDRs and the general conformational features of IDP ensembles [125–127]. Results from NMR, single-molecule fluorescence and computational approaches have shown that the charge features of IDPs influence the shape of their dynamic ensembles. Pappu and co-workers have extended these finding using both computational and experimental methods to show that not only the faction of charged residues and net charge per residue within IDPs and IDRs influence their overall conformational features, but also the distribution of oppositely charged residues within sequences significantly influences the compaction of IDP ensembles [128]. These advances have led to the development of a novel phase diagram based upon net positive and negative charge per residue values for the classification of IDP and IDR sequences [129]. These developments provide a conceptual framework for establishing relationships between the charge features of IDPs and IDRs, their conformational features and their propensities for phase separation. Charge features are certainly important factors governing protein phase separation behavior; for example, arginine residues are prevalent in low complexity regions known to form liquid-like droplets *in vitro* and within protein components of membrane-less organelles [44, 47]. However, these sequences are often enriched in aromatic and other neutral amino acids, indicating that, while electrostatic interactions may play important roles in some cases, other types of molecular interactions are at play in other cases [48, 50, 53]. This was born out in a recent study by García Quiroz and Chilkoti [130] in which they identified the sequence features of designed proteins that can undergo phase separation due to either a temperature increase (termed LCST sequences) or decrease (termed UCST sequences). The LCST sequences were enriched in hydrophobic residues while the UCST sequences were enriched in charges residues [131]. This study, which involved theoretical considerations as well as *in vitro* experimental measurements, serves as a model for future studies into the physical basis for phase separation of the growing list of proteins and RNA molecules shown to partition into the liquid-like or gel-like phase of membrane-less organelles and other cellular bodies.

#### Integrative approaches to understand the molecular basis of phase separation

None of the individual methods or approaches discussed above will alone uncover the molecular basis for phase separation by proteins and protein-nucleic acids mixtures; therefore, there is a need to apply multiple, complementary methods and to integrate results to advance mechanistic understanding. Integration is needed to span the broad length scales relevant to membrane-less organelles, ranging from the atomic scale (units of Å) relevant to amino acid conformations and their inter-molecular interactions to the overall size of *in vitro* liquid-like droplets and cellular membrane-less organelles (units of micrometers). Integration is also needed across the broad range of relevant time scales, including motions of amino acids and their polypeptide chains that mediate their conformational heterogeneity and inter-molecular interactions on the ns to μs time scale, to the diffusion of macromoclecules into and out of, and within, liquid-like structures on the timescale of seconds to tens of seconds. A key challenge is to understand the relationships between conformational features and motions of amino acids at the atomic scale and the macroscopic properties of these structures (e.g., viscosity, surface tension, macromolecular diffusion rates, etc.).

A few studies have begun to address the challenges associated with spanning these broad length and time scales. For example, a recent report addressed the conformational features of the FG-Nup protein, Nup153, and how these features mediate ultra-fast interactions the nuclear transport receptor, Importin β [132]. While not related to phase separation *per se*, this study provides an explanation for how Importin β-bound cargo can rapidly diffuse through the condensed phase within the core of the nuclear pore complex, which is comprised of several FG-Nup proteins, including Nup153. NMR spectroscopy was used to understand the ensemble averaged conformational and dynamic features of backbone amide groups within disordered Nup153 in the absence and presence of Importin-β and to generate a conformational ensemble using the sample-and-select approach. This ensemble was validated by back-calculation of the X-ray scattering profile and comparison with experimental SAXS data, an illustration of spanning length scales from amino acids to a whole disordered protein. To complement this information, data from smFRET and fluorescence lifetime measurements were used to understand the conformational features of many individual molecules under the same conditions while fluorescence correlation spectroscopy was used to compare molecular diffusion properties of Nup153 without and with Importin β. Additionally, molecular dynamics and Brownian dynamics computational methods were used to relate insights from the aforementioned biophysical methods to the mechanism of Nup153/Importin β interaction at atomistic resolution. Finally, these various pieces of molecular data were related to the Importin-β-dependent transport through the NPCs in live cells using bulk and single-particle fluorescence tracking.

Another example is provided by a recent study of the ALS-associated protein, FUS, from Fawzi and co-workers that employed NMR and various fluorescence microscopy methods to study the molecular features of FUS within *in vitro* liquid-like droplets and its interactions with RNA and the C-terminal domain of RNA Pol II. A final example is provided by a recent study of the highly abundant nucleolar protein, NPM1, which was shown to phase separate into liquid-like droplets with other nucleolar proteins and ribosomal RNA [NPM1 integrates within the nucleolus via multi-modal interactions with proteins displaying R-rich linear motifs and rRNA: Mitrea DM, et al., under review]. NMR, smFRET, and SANS were used to understand the conformational and dynamic features of NPM1 before and after phase separation with a peptide derived from the ribosomal protein, rpL5, and revealed molecular organization extending to ~10 nm within liquid-like droplets. In addition, deletion analyses identified the domains of NPM1 required for phase separation *in vitro* and for localization within nucleoli in cells.

The three studies discussed above illustrate approaches to relate the molecular features of phase-separation-prone proteins studied with atomic resolution to the macroscopic features of the liquid-like structures that they form. Importantly, two of the studies also integrated results from cellular assays, allowing molecular features to be related to biological function. We are just beginning to understand the physical properties of phase separation-prone proteins that are associated with their localization within membrane-less organelles and eagerly await the results of similarly adventurous integrative studies to broaden our knowledge of these features and, importantly, how they contribute to the diverse biological processes that occur within liquid-like cellular bodies.

## Conclusions

The compartmentalization of macromolecules within living cells creates heterogenous functional assemblies that mediate diverse biological processes. Membrane-less organelle assembly follows the physical laws of polymer condensation and depends upon factors such as component concentration and temperature (Fig. 1). Condensation is triggered by specific, initiating interactions between multivalent macromolecules and is further extended by recruitment of additional protein or RNA molecules via monovalent or multivalent interactions (Fig. 2). The complex composition of the intra-organelle matrix arises and is maintained by weak, multivalent interactions between modular proteins and RNA.

Condensation through phase separation of specific proteins and nucleic acids into dense liquid- or gel-like structures increases the local concentration of components involved in particular functions, possibly to optimize biochemical processes such as substrate-to-enzyme transfer. The concentration threshold for phase separation can be tuned by modulating the affinity of the interactions that promote phase separation (e.g., through posttranslational modification of proteins), thus altering the concentration of macromolecules in free solution. This “tuning” of phase separation behavior controls the participation of components of membrane-less organelles in stress signaling pathways (Fig. 2).

A deeper understanding of the multifarious, collective molecular interactions that promote condensation of membrane-less organelles and their functional roles in signal transduction under normal and stress conditions will empower the development of novel pharmaceutical agents to treat diseases in which the function of membrane-less organelles is altered, such as in cancer, neurodegenerative diseases and viral infections. A new branch of integrative structural biology is emerging, for which the challenges are to understand the *structural and dynamic* bases of phase separation in reconstituted *in vitro* systems as well as within intact cellular bodies and the relationships between these features and the biological processes that occur within membrane-less organelles. Based on new developments in the field, exciting opportunities for therapeutically targeting the meta-stable structural states of membrane-less organelles to modulate their signaling behavior are on the horizon.

## Abbreviations

mRNP: messenger ribonucleoprotein
snRNP: small nuclear ribonucleoprotein
snoRNP: small nucleolar ribonucleoprotein
TEM: transmission electron microscopy
FC: fibrillar centers
DFC: dense fibrillar component
GC: granular component
rRNA: ribosomal RNA
rDNA: ribosomal DNA
RNA Pol I/II: RNA polymerase I/II
NOR: nucleolar organizing region
SAXS: small angle X-ray scattering
SANS: small angle neutron scattering
FRAP: fluorescence recovery after photobleaching
FLIP: fluorescence loss in photobleaching
smFRET: single molecule Förster resonance energy transfer
